# Disease-associated mutations in CNGB3 promote cytotoxicity in photoreceptor-derived cells

**DOI:** 10.1167/13.9.1268

**Published:** 2013-06-11

**Authors:** Chunming Liu, Tshering Sherpa, Michael D. Varnum

**Affiliations:** 1 Western University of Health Sciences, College of Optometry, Pomona, CA; 2 Department of Integrative Physiology and Neuroscience, Washington State University, Pullman, WA; 3 Program in Neuroscience, Washington State University, Pullman, WA; 4 Center for Integrative Biotechnology, Washington State University, Pullman, WA

## Abstract

**Purpose:**

To determine if achromatopsia associated F525N and T383fsX mutations in the CNGB3 subunit of cone photoreceptor cyclic nucleotide-gated (CNG) channels increases susceptibility to cell death in photoreceptor-derived cells.

**Methods:**

Photoreceptor-derived 661W cells were transfected with cDNA encoding wild-type (WT) CNGA3 subunits plus WT or mutant CNGB3 subunits, and incubated with the membrane-permeable CNG channel activators 8-(4-chlorophenylthio) guanosine 3′,5′-cyclic monophosphate (CPT-cGMP) or CPT-adenosine 3′,5′-cyclic monophosphate (CPT-cAMP). Cell viability under these conditions was determined by measuring lactate dehydrogenase release. Channel ligand sensitivity was calibrated by patch-clamp recording after expression of WT or mutant channels in Xenopus oocytes.

**Results:**

Coexpression of CNGA3 with CNGB3 subunits containing F525N or T383fsX mutations produced channels exhibiting increased apparent affinity for CPT-cGMP compared to WT channels. Consistent with these effects, cytotoxicity in the presence of 0.1 μM CPT-cGMP was enhanced relative to WT channels, and the increase in cell death was more pronounced for the mutation with the largest gain-of-function effect on channel gating, F525N. Increased susceptibility to cell death was prevented by application of the CNG channel blocker L-*cis*-diltiazem. Increased cytotoxicity was also found to be dependent on the presence of extracellular calcium.

**Conclusions:**

These results indicate a connection between disease-associated mutations in cone CNG channel subunits, altered CNG channel-activation properties, and photoreceptor cytotoxicity. The rescue of cell viability via CNG channel block or removal of extracellular calcium suggests that cytotoxicity in this model depends on calcium entry through hyperactive CNG channels.

## Introduction

Rod and cone photoreceptor cells are highly specialized to carry out their primary task: transforming absorbed light into electrical responses that can be processed and understood as vision by the central nervous system. Long-term perturbations in components of the signal transduction cascade, energy metabolism, or structural integrity within the photoreceptors or their supporting cells can increase the risk of photoreceptor cell death (see reviews in [1-3]). The resulting loss of vision is one of the most common causes of disability. However, the exact cellular and molecular mechanisms by which mutations or environmental insults lead to photoreceptor cell death are not completely understood.

One critical component of the phototransduction cascade is the cyclic nucleotide-gated (CNG) channels in the outer segment plasma membrane of rods and cones. Closure of these channels converts the chemical signal (a fall in intracellular guanosine 3′,5′-cyclic monophosphate [cGMP] concentration) that is initiated by light absorption, into membrane hyperpolarization and decreased neurotransmitter release onto second-order cells (reviewed in [4]). The specialized CNG channels of cone photoreceptors are composed of CNGA3 and CNGB3 subunits in a two plus two configuration around the central pore [5] (but see also [6]). Mutations in the genes encoding these subunits have been linked to complete and incomplete achromatopsia [7-17], progressive cone dystrophy [11,18], macular degeneration, and macular malfunction [14]. Recent studies have determined how several disease-associated mutations in CNGA3 [15,19-24] and CNGB3 [25-27] subunits alter the functional properties of recombinant cone CNG channels, but the possible cellular consequences of these mutations are not well understood. For CNGA3 mutations, many have been shown to produce loss-of-function changes such as misfolding, intracellular retention, and/or reduced sensitivity to ligands. Recently, trafficking defective CNGA3 subunits bearing select disease-linked mutations were shown to produce endoplasmic reticulum (ER) stress, activation of the unfolded protein response, and decreased cell viability [28]. Similarly, in CNGA3-deficient (*CNGA3*^−/−^) mice, cones exhibit altered trafficking and/or expression levels for various proteins involved in the phototransduction cascade and apoptotic cell death [29].

Several mutations in CNGB3 have gain-of-function effects on channel gating [25,26], producing CNG channels that are more sensitive to cGMP. How these gain-of-function changes in CNG channel gating may lead to cone dysfunction and degeneration is a question that has not yet been addressed. Since CNG channels are the main pathway for Ca^2+^ entry into the outer segment of photoreceptors [30,31], we hypothesized that gain-of-function mutations in CNGB3 increase susceptibility to cell death via a Ca^2+^ overload mechanism. To address this issue, we have used photoreceptor-derived 661W cells as an in vitro model to investigate the effect of CNG channel mutations on cell viability. These cells exhibit many of the cellular and biochemical features of cone photoreceptor cells [32-34], but are reported to lack endogenous CNGA3 subunits [35]. Our experimental approach was to compare the viability of cells expressing wild-type (WT) or mutant CNG channels, measured primarily using lactate dehydrogenase (LDH) release as a reporter for cell death, after exposure to physiologically relevant concentrations of the membrane permeable channel activators 8-(4-chlorophenylthio) (CPT)-cGMP and/or CPT- adenosine 3′, 5′-cyclic monophosphate (cAMP). In this study, we have found that two mutations in CNGB3, which were linked previously to achromatopsia, progressive cone dystrophy, and/or macular degeneration, increased susceptibility to cell death. The increase in cytotoxicity associated with activation of mutant CNG channels was alleviated by the application of the CNG channel blocker or the removal of extracellular Ca^2+^. The results imply a connection between the altered gating properties of mutant CNG channels and photoreceptor cell death, providing insight into the cellular and molecular mechanisms underlying inherited retinal degeneration.

## Methods

### Molecular biology

Expression constructs for WT or mutant human CNGA3 and CNGB3 subunits in the vector pGEMHE were generated as described previously [26]. For expression in mammalian cells, cDNAs for CNGA3 or CNGB3 were subcloned into the pOPRSVI vector (Stratagene, La Jolla, CA) using unique restriction sites. The QuikChange® II Site-Directed Mutagenesis kit (Stratagene) was then used to generate point mutations in CNGB3. All mutations were confirmed by DNA sequencing.

### Functional expression in Xenopus laevis oocytes

For heterologous expression in Xenopus laevis oocytes, identical amounts of cDNA were linearized using *Sph*I or *Nhe*I, and capped cRNA was transcribed in vitro using the T-7 RNA polymerase mMESSAGE mMACHINE® kit (Ambion, Austin, TX). cRNA concentrations and relative amounts were determined by denaturing gel electrophoresis and KODAK 1D image analysis software (Rochester, NY), as well as by spectrophotometry. Oocytes were isolated as previously described [36] and microinjected with a fixed amount of cRNA for all constructs (approximately 5 ng of CNGA3 and 20 ng of CNGB3, a ratio shown previously to efficiently generate heteromeric channels [5]). Oocytes were incubated in ND96 (96 mM NaCl, 2 mM KCl, 1.8 mM CaCl_2_, 1 mM MgCl_2_, and 5 mM HEPES, pH 7.6, supplemented with 10 μg/ml gentamycin).

### Electrophysiology

Two to seven days after microinjection of cRNA, patch-clamp experiments were performed using the inside-out configuration with an Axopatch 200B amplifier (Axon Instruments, Foster City, CA). Recordings were made at 20–23 °C. Data were acquired using Pulse software (HEKA Elektronik, Lambrecht, Germany). Current traces were elicited by voltage steps from a holding potential of 0 mV to +80 mV, then to −80 mV and back to 0 mV. Initial pipette resistances were 0.4–0.8 megaohms. Intracellular and extracellular solutions contained 130 mM NaCl, 0.2 mM EDTA, and 3 mM HEPES (pH 7.2). Intracellular solutions were exchanged using an RSC-160 rapid solution changer (Molecular Kinetics, Indianapolis, IN). Currents in the absence of cyclic nucleotides were subtracted. For channel activation by CPT-cGMP or CPT-cAMP, dose–response data were fitted with the Hill equation, *I*/*I*_max_=([cNMP]*^h^*/(*K_1/2_^h^* + [cNMP]*^h^*)), where *I* is the current amplitude at +80 mV, *I*_max_ is the maximum current elicited by saturating concentration of ligand, [cNMP] is the ligand concentration, *K*_1/2_ is the apparent ligand affinity, and *h* is the Hill slope. We measured sensitivity to block by L-*cis*-diltiazem (RBI, Natick, MA) applied to the intracellular face of the patch in the presence of 0.1 μM CPT-cGMP. Data were fit with a modified Hill equation in the form *I*_blocker_/*I*=(*K_1/2_^h^*/(*K_1/2_^h^* + [blocker]*^h^*)). Data were analyzed using Igor (Wavemetrics, Lake Oswego, OR), SigmaPlot, and SigmaStat (Systat Software Inc., San Jose, CA). All values are reported as the mean±standard error of the mean of *n* experiments unless otherwise indicated. Statistical significance was determined using a Student *t* test or Mann–Whitney rank sum test, and a p value of <0.05 was considered significant.

### Cell culture and transfection of cDNAs

The mouse photoreceptor 661W cell line used in this study was generously provided by Dr. Al-Ubaidi (University of Oklahoma Health Sciences Center, Oklahoma City, OK). The 661W cells were routinely maintained in Dulbecco’s modified Eagle’s medium (Gibco, Carlsbad, CA), supplemented with 10% fetal bovine serum (Gemini Bioproducts, Sacramento, CA) and 1% penicillin/streptomycin (Gibco), at 37 °C in a humidified incubator with 5% CO_2_; cells were subcultured every 3–5 days. The 661W cells were transfected with pOPRSVI plasmids encoding human cone CNG channel subunits using Lipofectamine™ 2000 and OptiMEM (Life Technologies, Carlsbad, CA) according to the manufacturer’s protocol for cells in suspension. A reporter plasmid—a green fluorescent protein-expressing vector (pQBI25-fC2, Wako Pure Chemical Industries, Ltd., Japan) using a constitutive CMV promoter—was transfected under the same conditions to assess transfection efficiency. Transfection efficiencies of greater than 70% of cells were routinely observed. In addition, the pOPRSVI plasmid was transfected alone as a negative control. The amounts of each vector were as follows (µg/10 cm^2^ culture surface): 2 FLAG- or GFP-CNGA3 plus 2 FLAG-CNGB3 (WT, T383fsX or F525N); 4 GFP; or 4 pOPRSVI.

### Immunoblotting

Western blot analysis of proteins from 661W cells transfected with FLAG-tagged WT and mutant cone CNG channel subunits was performed. Cells were gently rinsed with PBS, scraped and lysed into cell lysis buffer containing 20 mM HEPES (pH 7.5), 150 mM NaCl, 5 mM EDTA, 0.5% Triton X-100 (Surfact-Amps X-100; Pierce Biotechnology, Rockford, IL), and a protease inhibitor cocktail (Complete™ Mini EDTA-free; Roche Applied Science, Indianapolis, IN). Samples were run under reducing conditions using NuPAGE® LDS Sample Buffer and Reducing Agent (Life Technologies). Samples were centrifuged for 2 min at 10,000 × g to collect insoluble material. Proteins were separated by SDS-PAGE using 4%–12% Bis-Tris NuPAGE® gels in MES/SDS Running Buffer plus Antioxidant (Life Technologies), then transferred onto nitrocellulose membranes using the NuPage® Transfer Buffer (Life Technologies). Immunoblots were probed with monoclonal anti-FLAG M2 antibody (Sigma-Aldrich, St. Louis, MO) and processed using chemiluminescent detection as previously described [19]. To verify that approximately equal amounts of total protein were loaded in each lane, the same blots were probed with MAB1501 pan-actin antibody (Millipore, Temecula, CA).

### Cell viability assays

For most experiments, the LDH Cytotoxicity Detection Kit (Roche Applied Science) was used according to the manufacturer’s protocol (see also [37]). Briefly, cultured 661W cells were transfected with the desired plasmid constructs as described above and then plated in 96-well tissue culture plates at a density of approximately 8×10^3^ cells/well. Forty-eight hours after transfection, cells were treated with various concentrations of CPT-cGMP and/or CPT-cAMP (Sigma-Aldrich) alone or together with L-*cis*-diltiazem (Enzo Life Sciences, Inc., Farmingdale, NY) in Dulbecco’s modified Eagle’s medium supplemented with 1% fetal bovine serum for 24 h at 37 °C. Following treatment, half of the culture medium was transferred to another 96-well plate and the LDH released into the culture medium was measured to assess the number of damaged/dead cells. Cells in the original plate were then lysed and the total amount of cellular LDH was assessed. The percentage cytotoxicity was then calculated from the ratio of LDH concentration in the medium/cells, and was normalized to the percentage cytotoxicity in untreated cells transfected with control pOPRSVI plasmid only.

The viability of 661W cells transfected with WT or mutant CNG channels was also assessed using the LIVE⁄DEAD® Viability⁄Cytotoxicity Kit (Life Technologies) according to the manufacturer’s protocol. Briefly, cells were transfected under the conditions described above and plated into 4-well multi-chamber Lab-Tek glass slides (Nalge Nunc International, Rochester, NY). At the end of cyclic nucleotide treatment, cells were washed with PBS three times and stained with 2 μM calcein AM and 4 μM ethidium homodimer-1 solution at room temperature for 30 min. Fluorescence microscopy was then performed to visualize the live and dead cells. Imaging of cells was performed at the Washington State University Franceschi Microscopy and Imaging Center. Images were obtained using a 10× objective on an Axiovert 200M inverted microscope equipped with a Zeiss LSM 510 confocal laser-scanning system and a krypton-argon laser. Fluorescence was measured using an excitation wavelength of 488 nm, and a 522 DF 32 emission filter for green fluorescence and 635 DF 32 emission filter for red fluorescence.

### Annexin V staining

For examination of apoptosis, 661W cells were transfected with WT channels, mutant channels, or vector only. Transfected cells were grown on poly-L-lysine-coated glass coverslips at the same density as described above and 0.1 μM CPT-cGMP was applied 48 h post transfection. Cultures were stained with fluorescein-labeled annexin V according to the manufacturer’s instructions (Biotium Inc., Hayward, CA). Cells were subsequently stained with ethidium homodimer to identify necrotic cells (not shown in images), and with DAPI to facilitate cell counting. Cells that were annexin V positive but ethidium homodimer negative were counted. Cells were visualized using fluorescence microscopy (Leica, Wetzlar, Germany). The percentage of annexin V–positive cells was calculated by selecting a high-density area of each coverslip and counting all cells within the focal field (typically >100 cells). Measurements were performed on coded samples to avoid biasing the results. Each transfection was replicated four times.

### Statistics

All statistical analyses were performed using Igor (Wavemetrics) and SigmaStat (Systat Software Inc.), and expressed as mean±standard error of the mean. Statistical significance was determined using a Student *t* test, analysis of variance, or the Mann–Whitney rank sum test, and a p value of <0.05 was considered significant.

## Results

### Disease-associated mutations in CNGB3 increase channel ligand sensitivity

**Figure 1:**
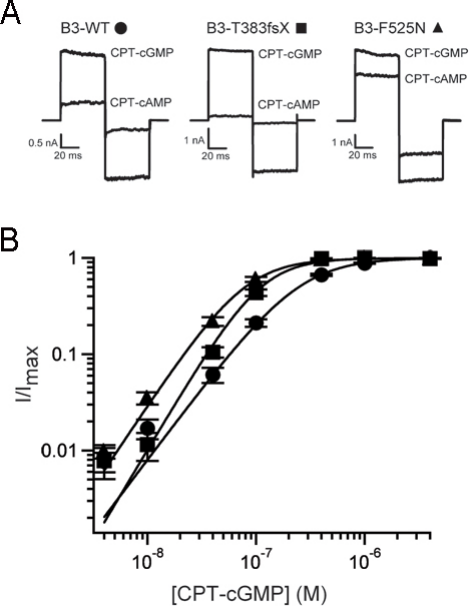
Disease-associated mutations in CNGB3 alter the gating properties of heteromeric channels. **A**: Representative current traces are shown for CNGA3 plus CNGB3 channels after activation by saturating concentrations of CPT-cGMP (4 μM) or CPT-cAMP (100 μM). Current traces were elicited by voltage steps from a holding potential of 0 mV to +80 mV, −80 mV, and then back to 0 mV. **B**: Representative dose–response relationships for CPT-cGMP activation of CNG channels, after expression of CNGA3 plus CNGB3-WT (circles), T383fsX (squares), or F525N (triangles) subunits. Currents were normalized to the maximum cGMP current. Continuous curves represent fits of the dose–response relationship with the Hill equation as described in the Methods section. The parameters for each channel type were as follows: for WT, *K*_1/2,CPT-cGMP_=248 nM, *h*=1.5; for T383fsX, *K*_1/2, cGMP_=111 nM, *h*=1.9; and for F525N, *K*_1/2,CPT-cGMP_=79 nM, *h*=1.7.

Disease-associated mutations in the CNGB3 subunit of cone CNG channels have been previously shown to produce channels with gain-of-function changes in channel-gating properties [25,26]. We investigated whether coexpression of mutant F525N or T383fsX CNGB3 subunits with WT CNGA3 subunits altered the sensitivity of the resulting channels to membrane-permeable analogs of cGMP and cAMP, CPT-cGMP, and CPT-cAMP. FLAG-tagged WT or mutant human CNGB3 subunits were heterologously expressed with WT human CNGA3 subunits in *X. laevis* oocytes. Patch-clamp recordings were performed with excised membrane patches using the inside-out configuration; channels were activated by the application of solutions containing cyclic nucleotides to the intracellular face of the membrane patch (Figure 1A). Many disease-associated mutations in CNGA3 subunits cause intracellular retention and reduced functional expression levels [15,19-24]. For the CNGB3 mutations investigated here, maximum patch current density (I_max_/area), determined at +80 mV in a saturating concentration of CPT-cGMP (4 μM), was not significantly altered by the mutations (WT: 55.1±10.9 pA/μm^2^, n=13; T383fsX: 86.6±17.0 pA/μm^2^, n=9; F525N: 80.3±18.6 pA/μm^2^, n=15). This indicates that the number of functional CNG channels in the plasma membrane was not reduced by these mutations.

We also determined the relative agonist efficacy for channel activation by a saturating concentration of CPT-cAMP compared with the maximal activation by CPT-cGMP (*I*_max, CPT-cAMP_/*I*_max, CPT-cGMP_). For photoreceptor CNG channels, cAMP is a partial agonist while cGMP is nearly a full agonist. Thus, changes in cAMP efficacy can report alterations in channel gating properties. Currents elicited at +80 mV by saturating concentrations of CPT-cGMP (4 μM) or CPT-cAMP (100 μM) revealed that CPT-cAMP is a partial agonist compared to CPT-cGMP, similar to the relationship between the natural agonists cAMP and cGMP. CNG channels containing the F525N mutation exhibited a significant increase in CPT-cAMP efficacy (*I*_max, CPT-cAMP_/*I*_max, CPT-cGMP_=0.49±0.04, n=15) compared to that of WT heteromeric channels (*I*_max, CPT-cAMP_/*I*_max, CPT-cGMP_=0.25±0.03, n=10; p=0.001); this increase in relative CPT-cAMP efficacy agrees with the increase in unmodified cAMP efficacy previously reported for F525N [25]. Channels formed after the expression of CNGB3 T383fsX with CNGA3 showed a significant decrease in CPT-cAMP efficacy (*I*_max, CPT-cAMP_/*I*_max, CPT-cGMP_=0.07±0.01, n=8; p<0.001; Figure 1A). Reduced CPT-cAMP efficacy with coexpression of CNGB3 T383fsX is consistent with the expected lack of functional CNGB3 subunits and the resulting generation of homomeric CNGA3-only channels [26]. For F525N-containing channels, increased CPT-cAMP efficacy reflects a gain-of-function change in channel gating.

Next, we determined the effect of the CNGB3 mutations on the CPT-cGMP sensitivity of the channels. The currents elicited by various concentrations of CPT-cGMP were measured at +80 mV. The apparent CPT-cGMP affinity (*K*_1/2, CPT-cGMP_) of WT and mutant channels was then determined from the dose–response relationships for channel activation, using fits with the Hill equation. Compared to WT heteromeric channels (*K*_1/2, CPT-cGMP_=254.0±18.1 nM, *h*=1.5±0.05, n=13), channels formed after expression of CNGB3 T383fsX with CNGA3 subunits were more sensitive to CPT-cGMP (*K*_1/2, CPT-cGMP_=105.1±7.3 nM, *h*=2.2±0.1, n=8; p<0.01; Figure 1B). T383fsX likely represents a functional null mutation, producing only homomeric CNGA3 channels at the plasma membrane [26]. Consistent with this idea, homomeric CNGA3-only channels exhibited a similar apparent affinity for CPT-cGMP (*K*_1/2, CPT-cGMP_=113.9±7.9 nM, *h*=2.2±0.1, n=9; data not shown). Channels containing CNGB3-F525N exhibited a larger increase in apparent ligand affinity (*K*_1/2, CPT-cGMP_=86.8±10.6 nM, *h*=1.8±0.04; p<0.01; Figure 1B), in agreement with previous studies using unmodified cGMP [25]. Overall, 8-(4-chlorophenylthio)-modified cGMP was 80–100 fold more potent than unmodified cGMP [19,26] for activation of human CNGA3 plus CNGB3 channels, similar to its increased potency observed with rod CNG channels [38,39]. These results illustrate the functional disturbances produced by F525N or T383fsX mutations, and help calibrate the physiologically appropriate CPT-cGMP concentration range for cone CNG channel activation.

### Disease-associated mutations in CNGB3 increase susceptibility to cell death in photoreceptor-derived cells

**Figure 2:**
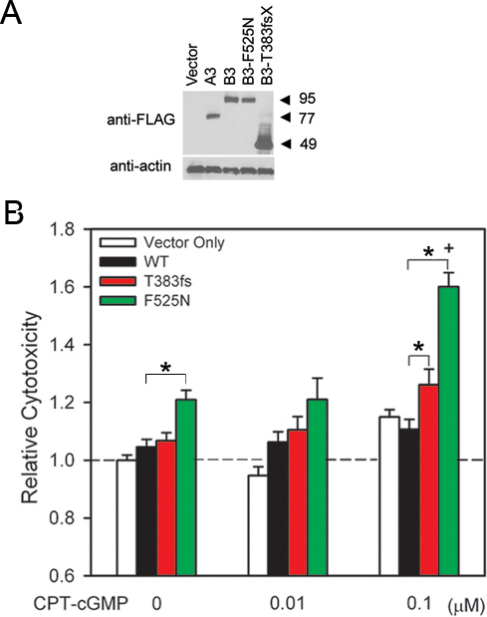
Disease-associated mutations in CNGB3 increase cytotoxicity. **A**: Western blot demonstrating expression of FLAG-tagged wild-type (WT) and mutant cone CNG channel subunits in 661W cells following transfection with indicated plasmids (above). Approximate locations of molecular weight markers (in kilodaltons) are indicated to the right of the immunoblot. Cell lysates were also probed with beta-actin antibody (below). The molecular weight of beta actin was ~42 kDa. **B**: The bar graph demonstrates increased cytotoxicity (measured as LDH release from dying cells) for cells expressing CNGA3 plus wild-type (WT) or mutant CNGB3 exposed to various concentrations of CPT-cGMP for 24 h (n=46 to 48). Cytotoxicity was normalized to that of control cells transfected with vector (pOPRSVI) alone, incubated in the absence of CPT-cGMP; *, significant difference between groups indicated by bracket (p<0.05); +, significant difference between F525N groups with or without 0.1 μM CPT-cGMP treatment (p<0.01).

We used a cone photoreceptor derived cell line (661W) to investigate the possible effects of mutant CNG channels on cell viability. These 661W cells represent a well-established model for photoreceptor cell death studies [33,40-43], and have been shown to express several markers characteristic of cone but not rod photoreceptors [32]. First, we confirmed the expression of FLAG-tagged CNGA3 or CNGB3 in 661W cells via immunoblotting, after transfection of plasmids encoding WT or mutant channel subunits (Figure 2A). Constructs expressing WT CNGA3 or CNGB3, or CNGB3 subunits containing F525N or T383fsX mutations all produced robust protein levels for the respective subunits. As described previously [26], the T383fsX mutation generated severely truncated CNGB3 subunits having a molecular weight of approximately 47 kDa, compared to ~74 kDa and ~95 kDa for WT CNGA3 and CNGB3, respectively (Figure 2A).

To test the effect of the mutations on cell viability, cells were transiently transfected with WT or mutant CNGB3 subunits together with CNGA3 subunits, and treated with CPT-cGMP for 24 h at concentrations ranging from 0.01 µM to 10 µM. An LDH-release assay was then performed to assess cytotoxicity induced by activation of CNG channels. The results in each experiment were expressed as relative cytotoxicity normalized to percent cytotoxicity of untreated cells transfected with the pOPRSVI vector alone (control). As summarized in Figure 2B, incubation of cells expressing mutant CNG channel subunits with 0.1 µM CPT-cGMP produced a significant increase in relative cytotoxicity (T383fsX: 1.26±0.05, p<0.05; F525N: 1.59±0.05, p<0.01) compared to WT channels (1.11±0.04). The magnitude of the increase in relative cytotoxicity for the different channel mutations was in the same rank order as the increase in channel ligand sensitivity (Figure 1B). Furthermore, channel activation by 0.1 µM CPT-cGMP roughly mimics the low physiologic concentration of cGMP in photoreceptors and the low level of basal channel activity [30,44]. In the absence of CPT-cGMP treatment, cells expressing channels containing the F525N mutation also exhibited a small increase in relative cytotoxicity (1.21±0.03) compared to WT channels under these conditions (1.05±0.03; p<0.01), suggesting that activation of F525N-containing channels by endogenous cGMP (and/or endogenous cAMP) increased susceptibility to cell death. No significant difference in relative cytotoxicity was observed at CPT-cGMP concentrations of 1 µM or greater (data not shown). Together, these results suggest a correlation between elevated CNG channel activity and cell death.

**Figure 3:**
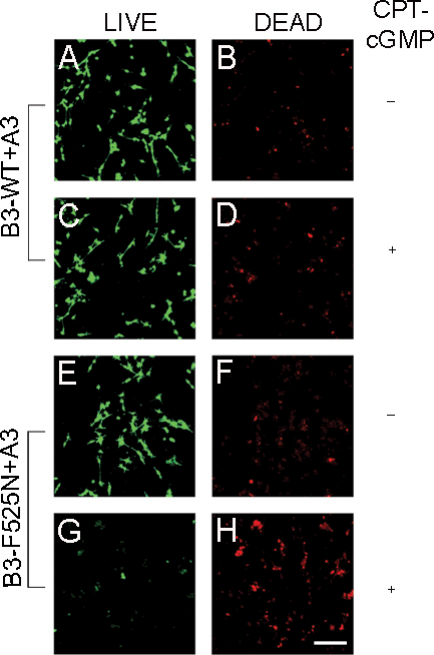
CNGB3 F525N mutation impairs cell viability with CPT-cGMP treatment. Transfected 661W cells were treated with or without 0.1 μM CPT-cGMP for 24 h. After treatment, cells were labeled according to the LIVE/DEAD assay protocol: vital cells were stained by calcein AM and show green fluorescence (**A**, **C**, **E**, and **G**); damaged cells were penetrated by ethidium homodimer and show red fluorescent nuclei (**B**, **D**, **F** and **H**). Fluorescent images were obtained using a Zeiss LSM 510 confocal laser-scanning microscope as described in the Methods section.

To confirm the effects of mutant CNG channel subunits on photoreceptor viability, we used an alternate assay (LIVE/DEAD Viability/Cytotoxicity Kit) to label live and dead cells. Cells were transfected with mutant or WT channels as described above; fluorescence microscopy was performed after 0.1 µM CPT-cGMP treatment, and calcein AM and ethidium homodimer-1 staining of the transfected cells. As shown in Figure 3, CPT-cGMP-treated cells expressing CNG channels with the F525N mutation exhibited more damaged/dead cells (red fluorescence) and less intact/viable cells (green fluorescence) compared to cells expressing WT channels or untreated F525N-expressing cells. In addition, a decrease in overall cell number was observed for CNGB3 F525N-expressing cells (Figure 3).

**Figure 4:**
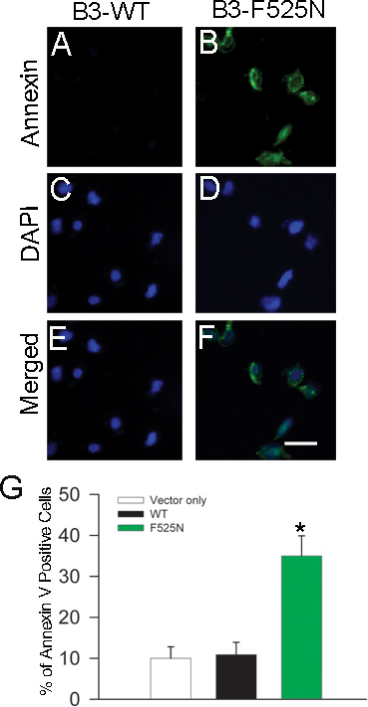
CNGB3 F525N mutation increases annexin V–positive cells compared to wild-type channels. Transfected cells were treated with 0.1 μM CPT-cGMP for 24 h. For determination of cell death, cells were stained with fluorescein-labeled annexin V, a protein with a high affinity for phosphatidylserine (**A** and **B**). Cells were also counterstained with DAPI to count nuclei (**C** and **D**), and the two images were merged to count annexin V–positive cells (**E** and **F**). **G**: Summary bar graph for annexin V staining of cells transfected with control plasmid, wild-type CNGB3 plus CNGA3, or CNGB3-F525N plus CNGA3 plasmids. Fluorescent images were obtained using a Zeiss LSM 510 confocal system as described in the Methods. Scale bar in **F** (applies to **A**-**F**), 100 µm.

Next, we used fluorescein-labeled annexin V, a protein with high affinity for phosphatidylserine, to test for potential apoptosis in F525N-expressing cells. Transfected cells grown on poly-L-lysine-coated glass coverslips were stained with annexin V (Figure 4A and B) and counterstained with DAPI to count the nuclei (Figure 4C and D). Images were merged (Figure 4 (E and F)) and cells were counted. As shown in Figure 4G, annexin V staining of cells transfected with CNGB3-F525N plus CNGA3 demonstrated significantly more annexin V–positive cells compared to control plasmid or WT CNGB3 plus CNGA3 (p<0.05).

### Low concentration of CPT-cAMP can have a protective effect on the viability of cells expressing CNGB3 F525N

**Figure 5:**
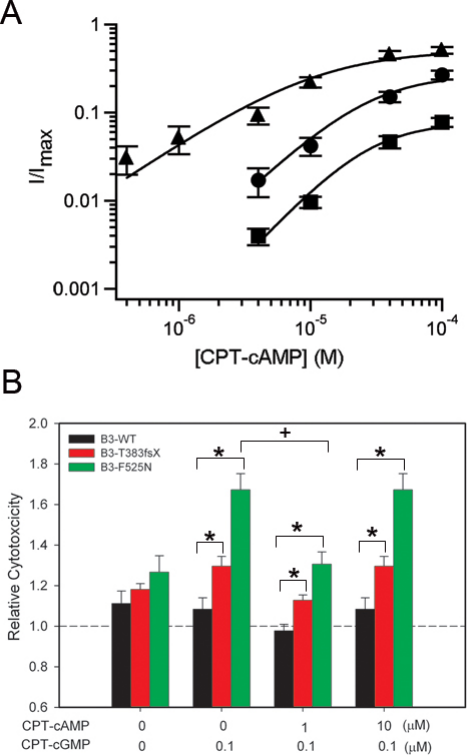
Effect of combined exposure to CPT-cAMP and CPT-cGMP on cytotoxicity of cells expressing channels with disease-associated mutations in CNGB3. **A**: Representative dose–response relationships for CPT-cAMP activation of CNG channels, after coexpression of CNGA3 with CNGB3-WT (circles), T383fsX (squares), or F525N (triangles) subunits (same representative patches as in Figure 1B). Currents were normalized to the maximum CPT-cGMP current. Continuous curves represent fits of the dose–response relationship with the Hill equation. Parameters for each channel type were as follows: for WT, *K*_1/2,CPT-cAMP_=28.3 μM and *h*=1.4; for T383fsX, *K*_1/2, cAMP_=27.9 μM and *h*=1.6; and for F525N, *K*_1/2,CPT-cAMP_=10.8 μM and *h*=1.0. **B**: Bar graph of the relative cytotoxicity for channel-expressing cells exposed to various concentrations of CPT-cAMP plus 0.1 μM CPT-cGMP (n=12). The dashed line represents the percentage of cell death in the vector-only control group without treatment; * indicates significant difference between groups designated by bracket (p<0.05); + represents significant difference between the F525N group treated with 10 μM CPT-cAMP together with 0.1 μM CPT-cGMP and the F525N group without treatment (p<0.01).

Although cGMP is the primary natural agonist for CNG channels in photoreceptors, both cAMP and cGMP coexist under physiological conditions. The level of intracellular cAMP also changes with illumination [45] and with photoreceptor degeneration [46]. In addition, photoreceptor cAMP concentration is controlled in a circadian manner, with higher levels at night [47]. We found that the F525N mutation produced a significant increase in apparent affinity for CPT-cAMP (*K*_1/2, CPT-cAMP_=13.8±1.4 µM, *h*=1.4±0.08; n=15) compared to WT channels (*K*_1/2, CPT-cAMP_=28.1±2.6 µM, *h*=1.7±0.1; n=10, p<0.01), while the T383fsX mutation produced no significant change in CPT-cAMP sensitivity (*K*_1/2, CPT-cAMP_=29.8±1.9 µM, *h*=1.8±0.1; n=9; Figure 5A).

Since CNG channels containing F525N exhibited a twofold increase in apparent cAMP affinity compared to WT channels (Figure 5A), we hypothesized that activation of mutant channels by CPT-cAMP might also increase cytotoxicity. Surprisingly, treatment of channel-transfected cells with CPT-cAMP (at concentrations ranging from 0.1 to 10 µM) produced no significant increase in relative cytotoxicity (data not shown). It has been demonstrated previously that cAMP and cGMP can have both synergistic and competitive interactions for CNG channel activation [48-50]. We applied CPT-cAMP and CPT-cGMP together to investigate the potential effect of this combination on cell viability. For F525N-containing channels, the coapplication of 1 µM CPT-cAMP with 0.1 µM CPT-cGMP attenuated the increased relative cytotoxicity induced by CPT-cGMP alone (CPT-cGMP alone: 1.59±0.05; CPT-cGMP with CPT-cAMP: 1.31±0.06, p<0.01; Figure 5B); differences in the relative cytotoxicity between WT (0.98±0.03) and mutant channels under these conditions remained statistically significant (p<0.01). As expected, relative cytotoxicity for cells expressing CNGB3 T383fsX was unaltered by coapplication of various concentration of CPT-cAMP compared to CPT-cGMP treatment alone. This likely reflects the lower CPT-cAMP efficacy (Figure 1A) and lower CPT-cAMP apparent affinity (Figure 5A) of CNGA3-only channels generated by the expression of CNGB3 T383fsX with CNGA3. Treatment with a higher concentration of CPT-cAMP (10 µM) combined with CPT-cGMP (0.1 µM) produced cytotoxicity for mutant channels comparable to that of 0.1 µM CPT-cGMP treatment alone (T383fsX: 1.30±0.05, p<0.01; F525N: 1.67±0.08; p<0.01, compared to WT channels). Together, these results show that a low concentration of CPT-cAMP when combined with CPT-cGMP can have a small protective effect on cell viability in the context of hyperactive F525N-containing channels. As CPT-cAMP is a less effective agonist relative to CPT-cGMP, this protection may be due to slight inhibition of channel activity compared to CPT-cGMP alone. Alternatively, protection may arise from some unknown, indirect pathway.

### Protective effects of CNG channel blocker or removal of extracellular calcium for cells expressing CNGB3 F525N

**Figure 6:**
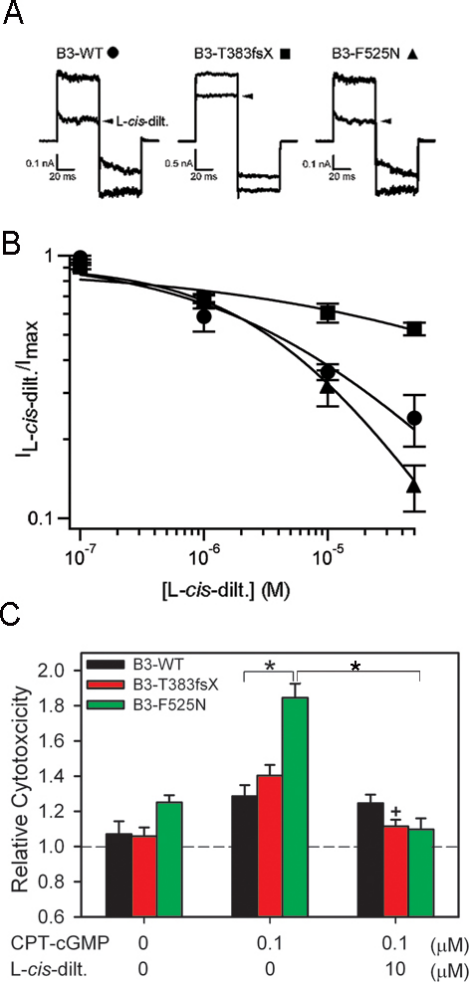
Block of CNG channels by L-*cis*-diltiazem increases viability of cells expressing CNGB3 with disease-associated mutations. **A**: Representative current traces elicited by 0.1 µM CPT-cGMP in the absence or presence (arrow) of 10 µM L-*cis*-diltiazem. Current traces were elicited by the voltage protocol described in the Methods section. **B**: Dose–response relationships for block by L-*cis*-diltiazem in the presence of 0.1 µM CPT-cGMP for heteromeric CNG channels containing CNGB3-WT (circles), T383fsX (squares), or F525N (triangles) subunits. Currents were normalized to the current elicited by 0.1 µM CPT-cGMP in the absence of L-*cis*-diltiazem. Continuous curves represent fits of the dose–response relation with the modified Hill equation described in the Methods section. Parameters for each channel type were as follows: WT, *K*_1/2,L-_*_cis_*_-dilt._=4.1 µM and *h*=0.5; for T383fsX, *K*_1/2,L-_*_cis_*_-dilt._=135 µM and *h*=0.3; and for F525N, *K*_1/2,L-_*_cis_*_-dilt._=4.2 µM and *h*=0.7. **C**: Bar graph of the relative cytotoxicity for channel-expressing cells with or without 10 µM L-*cis*-diltiazem in the presence of 0.1 μM CPT-cGMP treatment (n=12). The dashed line indicates the extent of cell death in vector-only control cells without treatment. Significant differences were observed between groups indicated by brackets (*, p<0.05); + indicates significant difference between T383fsX groups treated with or without channel blocker (p<0.01).

L-*cis*-diltiazem is a known CNG channel blocker that has been used extensively to dissect the properties of the native and heterologously expressed CNG channels [19,51-54]. In addition, some evidence suggests that diltiazem can protect photoreceptors from degeneration in the context of conditions producing elevated cGMP levels [55-57]. Since the increased cytotoxicity described above for CNGB3 mutations may be related to enhanced channel activity, we hypothesized that application of CNG channel blockers would exert a rescuing effect. We first used patch-clamp recordings to determine the sensitivity of WT and mutant channels to block by L-*cis*-diltiazem in the presence of CPT-cGMP. Figure 6A shows current recordings that illustrate block of CNG channels by 10 µM L-*cis*-diltiazem after channel activation by 0.1 µM CPT-cGMP. The apparent affinity for L-*cis*-diltiazem (*K*_1/2, L-_*_cis_*_-dilt._) was calculated by fitting dose–response relationships for channel block with a modified Hill equation, as described in the Methods section (Figure 6B). Compared to WT heteromeric channels (*K*_1/2, L-_*_cis_*_-dilt._=4.06±1.0 µM, n=5), F525N exhibited no significant change in apparent affinity for L-*cis*-diltiazem (*K*_1/2, L-_*_cis_*_-dilt._=3.03±0.75 µM, n=5). In contrast, T383fsX exhibited a large decrease in diltiazem apparent affinity (*K*_1/2, L-_*_cis_*_-dilt._=77.9±23.1 µM, n=4, p<0.01). Reduced sensitivity to block by diltiazem was in agreement with the previous finding that CNGB3 T383fsX prevents the formation of functional heteromeric channels, leading to CNGA3-only channels [26]. CNGA3-only channels are much less sensitive to block by L-*cis*-diltiazem compared to heteromeric channels composed of CNGB3 plus CNGA3.

We next examined the potential protective effects of CNG channel blockers on cell viability by applying 10 µM L-*cis*-diltiazem, in the presence of 0.1 µM CPT-cGMP, to transfected 661W cells. Application of L-*cis*-diltiazem effectively rescued cells from the increased cytotoxicity elicited by F525N channel activation (Figure 6C). Despite the reduced sensitivity to block by L-*cis*-diltiazem for T383fsX, the CPT-cGMP-induced increase in cytotoxicity was attenuated compared to no diltiazem treatment (Figure 6C). Thus, diltiazem may reduce cytotoxicity for T383fsX-expressing cells via some other mechanism independent of CNG channel block. For F525N-containing channels, rescue by L-*cis*-diltiazem is consistent with a link between cytotoxicity and active (open) CNG channels.

**Figure 7:**
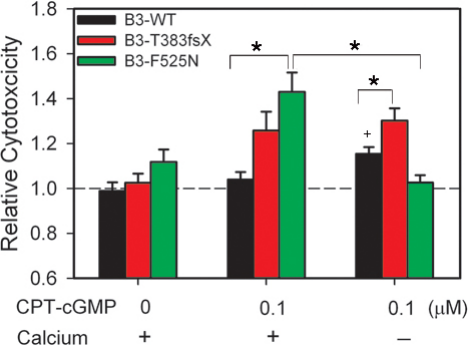
Removal of extracellular Ca^2+^ from culture media prevented the increase in cytotoxicity for cells expressing CNGB3 F525N. Bar graph of the relative cytotoxicity for channel-expressing cells treated with 0.1 μM CPT-cGMP in normal or Ca^2+^-free Dulbecco’s modified Eagle’s medium media (n=12; *, p<0.05). The dashed line indicates the level of cytotoxicity in control cells expressing pOPRSVI plasmid alone cultured in normal media without CPT-cGMP.

CNG channel mutations that increase ligand sensitivity are expected to increase calcium entry through the hyperactive, calcium-permeable channels. Calcium overload is thought to serve as an important trigger for photoreceptor degeneration [58]. Thus, we predicted that the increased cytotoxicity observed with CNGB3-F525N and T383fsX mutations would depend on extracellular calcium. To test this prediction, we assessed the relative cytotoxicity of transfected cells maintained in normal or Ca^2+^-free media during CPT-cGMP treatment. As shown in Figure 7, removal of extracellular Ca^2+^ effectively attenuated the increased relative cytotoxicity elicited by F525N-containing channels compared to WT channel activation. Removal of extracellular Ca^2+^ produced no significant change in cytotoxicity for vector-only control cells in the absence of CPT-cGMP (p=0.190; data not shown). In addition, the absence of extracellular Ca^2+^ had no significant effect on the relative cytotoxicity of cells expressing T383fsX subunits. These results suggest that other mechanisms independent of plasma membrane calcium entry might contribute to the T383fsX-induced increase in cytotoxicity. For example, active CNGA3-only channels may still enhance sodium entry, depolarization, and subsequent ATP depletion due to increased Na^+^/K^+^-exchanger activity [59,60]. Interestingly, removal of extracellular Ca^2+^ for cells expressing WT channels caused a mild increase in cytotoxicity (p=0.0115). Together, these results show that the increase in cytotoxicity arising from the activation of F525N-containing channels, but not CNGA3-only channels in T383fsX-expressing cells, depends on extracellular calcium.

## Discussion

We have examined the cellular consequences of two different *CNGB3* mutations linked to achromatopsia, cone dystrophy, and/or macular degeneration in humans. Our results indicate that the expression of CNGB3 subunits containing F525N or T383fsX mutations significantly increase susceptibility to cell death compared to WT channels in the presence of a low, physiologically relevant concentration of membrane-permeable channel activator. Higher levels of CPT-cGMP are expected to activate other cellular pathways, including those known to be neuroprotective [61]. However, the mutations permit an influx of Ca^2+^ at CPT-cGMP levels that are too low to trigger these neuroprotective mechanisms, and therefore cytotoxicity results. Importantly, the concentration of channel activator producing this difference in cytotoxicity was within the range showing the greatest difference between mutant and WT channel activation. This concentration of channel activator also mimics the low level of channel ligand and corresponding low level of channel activity existing in the photoreceptor outer segment in the dark [62-64]. In addition, increased susceptibility to cell death was prevented by a CNG channel blocker or by removal of extracellular calcium, consistent with the idea that photoreceptor death can arise via excess calcium entry through hyperactive CNG channels. Together, these results imply a connection between mutations in cone CNG channels, altered channel-activation properties, and cell death. This study highlights the critical role that CNG channels play in some forms of retinal degeneration, consistent with the recent discovery that a CNG channel knockout (CNGB1−/−) can rescue rod photoreceptors in *rd1* mice [65].

Drugs (e.g., L-*cis*-diltiazem) or other manipulations that block or inhibit photoreceptor CNG channel activity have potential therapeutic merit in the context of mutations that produce hyperactive CNG channels or elevated cGMP levels. One intriguing approach that has been proposed for hyperactive cone CNG channels is to adjust channel ligand sensitivity to normal levels via inhibition of gating with retinoids [66]. Similarly, augmented CNG channel inhibition via calmodulin or phosphoinositides might protect photoreceptors with hyperactive channels or elevated cGMP. Other alternatives include neuroprotection via growth factor receptor activation; targeting downstream effectors involved in cell death pathways; or enhanced calcium extrusion from photoreceptor outer segments [59,67].

The CNGB3 F525N mutation is located within the cytoplasmic C-linker region, which connects the CNBD to the pore-forming domain and participates in the conformational changes that covert ligand binding into channel opening. The phenylalanine at this position is highly conserved across CNG channels and related hyperpolarization-activated cyclic nucleotide-regulated (HCN) channels. The crystal structure of the C-terminal domain of homologous HCN2 channels [68] provides potential insight into the structural changes that may arise from the F525N substitution in CNGB3. The HCN2 structure is thought to represent a compact ligand-bound but closed conformation [69,70]. The residue in HCN2 (F518) that aligns with F525 in CNGB3 resides in the F’ helix and appears to be buried in this C-linker closed conformation [68]. The F’ helix of HCN2, in concert with the C helix of the CNBD, has been shown recently to be part of a key conformational rearrangement that occurs upon ligand binding and is proposed to help propagate the gating transition through the C-linker region to the pore [71]. The phenylalanine to asparagine substitution in CNGB3 reduces both side-chain volume and hydrophobicity. Consistent with a structure/function analogy to HCN2, one plausible interpretation for the effect of F525N on CNG channel gating is that it enhances channel activity via the destabilization of the closed-channel conformation.

We expect that in native photoreceptors of patients with gain-of-function CNG channel mutations such as F525N, the channels will have a higher probability of being open in the dark and fail to close appropriately during light stimulation. Because CNG channels are the primary entryway for calcium into the photoreceptor outer segment [30], hyperactive channels are expected to disturb calcium homeostasis in these cells. We hypothesize that abnormally high levels of calcium under these circumstances will lead to photoreceptor death. Similar cellular mechanisms have been described for mutations in genes encoding other critical proteins involved in phototransduction, adaptation, and recovery processes. Mutations that produce constitutively active guanylyl cyclase [72-74] or loss of cGMP-phosphodiesterase activity [75-77] result in increased intracellular cGMP levels. Increased intracellular cGMP, similar to an increase in channel sensitivity to cGMP, is expected to lead to inappropriate opening of the channels, with more Ca^2+^ entering the photoreceptor. Numerous studies have reported that a sustained elevation of intracellular Ca^2+^ can result in apoptotic cell death (reviewed by Choi [78] and Leist and Nicotera [79]). In the retina, for example, the elevation of intracellular Ca^2+^ has been shown to trigger rod photoreceptor apoptosis and retinal degeneration [80]. Some of the Ca^2+^-dependent pathways producing photoreceptor degeneration are thought to involve caspase and/or calpain activation as a central mechanism [57,81,82], but some diversity of cell death mechanisms, including autophagy, has also been reported [58,60,83,84]. The exact intracellular pathways involved in photoreceptor degeneration caused by cone CNG channel mutations remain to be determined.

For the F525N mutation, our results strongly suggest that channel hyperactivity and subsequent Ca^2+^ overload promote an increase in cell death. However, other possible mechanisms may contribute to cytotoxicity in the context of CNGB3 mutations. In particular, the cellular consequences of T383fsX are more difficult to interpret. This frameshift is effectively a null mutation, producing a truncated CNGB3 subunit that does not combine with CNGA3 subunits to form functional heteromeric channels at the plasma membrane [26]. Homomeric CNGA3-only channels exhibit greater apparent affinity for cGMP compared to heteromeric channels, a gain-of-function phenotype. In addition, homomeric CNGA3 channels are insensitive to downregulation by Ca^2+^/calmodulin [85] or by phosphoinositides such as PIP_3_ [86,87]. It is possible that lack of proper regulation of the channels may contribute to enhanced channel activity and increased susceptibility to cell death. In addition, improperly localized [26] or misfolded CNGB3-T383fsX subunits may produce ER stress and induce the unfolded protein response, subsequently leading to cell death (see review in [88]). Similarly, we have demonstrated recently that disease-associated missense mutations in CNGA3 can produce ER stress/ unfolded protein response activation with a concomitant loss of cell viability [28]. Consistent with this potential mechanism, removal of extracellular Ca^2+^ did not significantly alleviate the effect of T383fsX on cell viability. It is also possible that the premature stop codon upstream of the intronic sequence in *CNGB3* transcripts induces nonsense mediated messenger RNA decay during processing of the T383fsX message in vivo. Cone CNG channel deficiency in mice, including CNGB3^−/−^, has been shown recently to lead to photoreceptor degeneration that is associated with ER stress, defective trafficking of outer segment proteins, and apoptosis [89]. Further studies are needed to address these alternative mechanisms.
